# Supplementary figures and images for: An improved fluorescent noncanonical amino acid for measuring conformational distributions using time-resolved transition metal ion FRET
Source: eLife. 2021 Oct 8;10:e70236. doi: 10.7554/eLife.70236 (PMC8500717; doi:10.7554/eLife.70236)

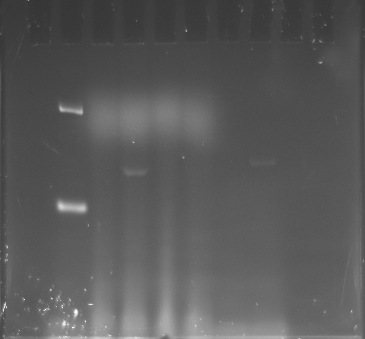

Supplement: Figure 2—source data 1. — Black arrow indicates the size of full-length MBP-295Acd. [file elife-70236-fig2-data1.tif]

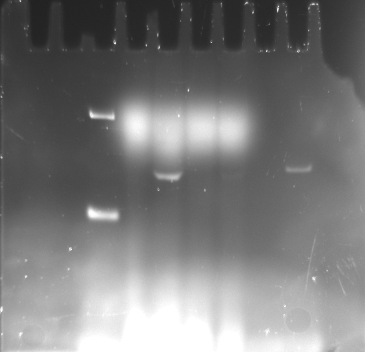

Supplement: Figure 2—source data 2. — Black arrow indicates the size of full-length MBP-322Acd. [file elife-70236-fig2-data2.tif]

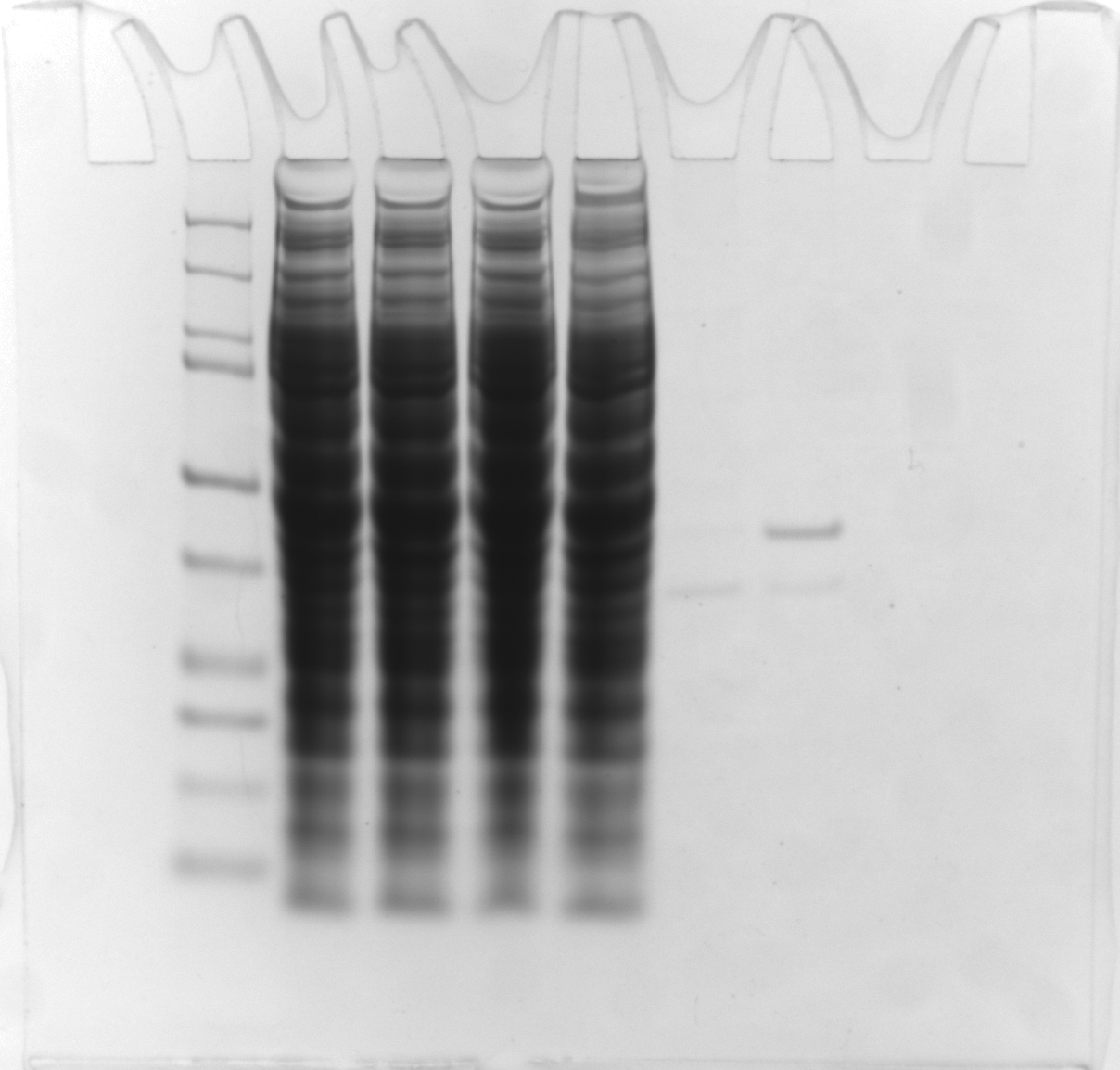

Supplement: Figure 2—source data 3. — Black arrow indicates the size of full-length MBP-295Acd, and red arrow indicates the size of the truncation product. [file elife-70236-fig2-data3.tif]

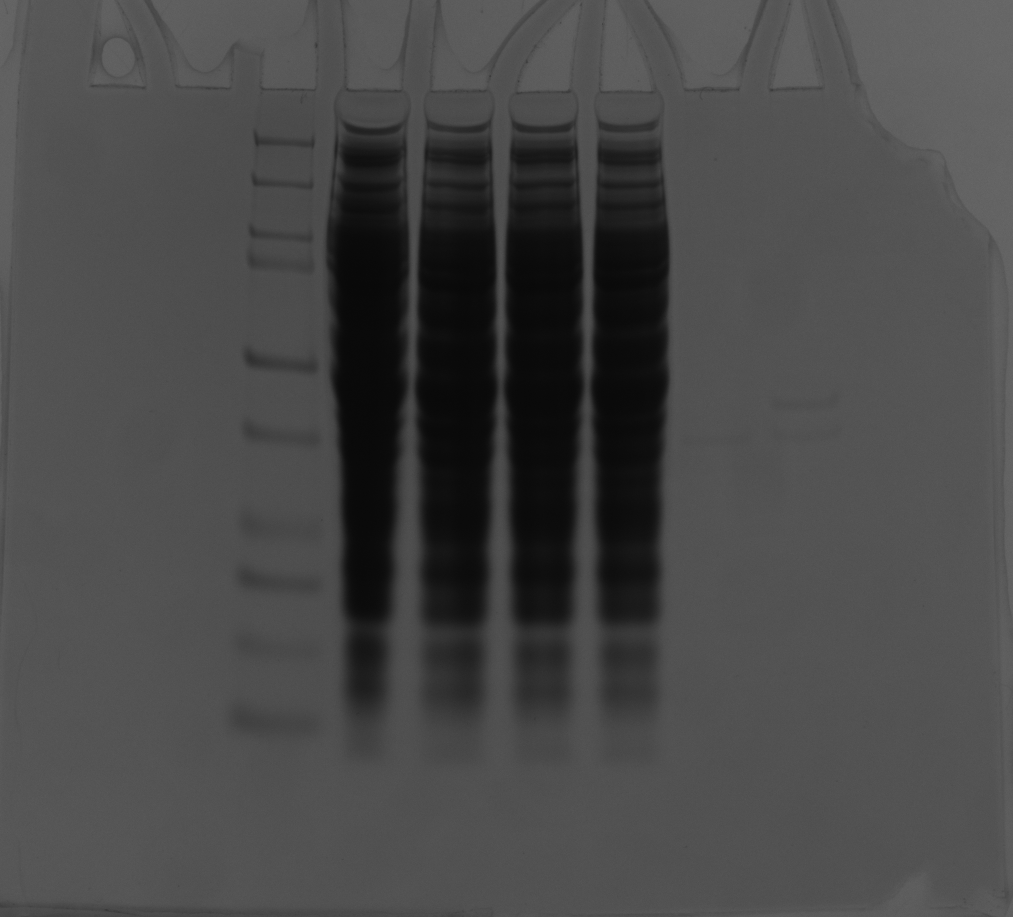

Supplement: Figure 2—source data 4. — Black arrow indicates the size of full-length MBP-322Acd, and red arrow indicates the size of the truncation product. [file elife-70236-fig2-data4.tif]

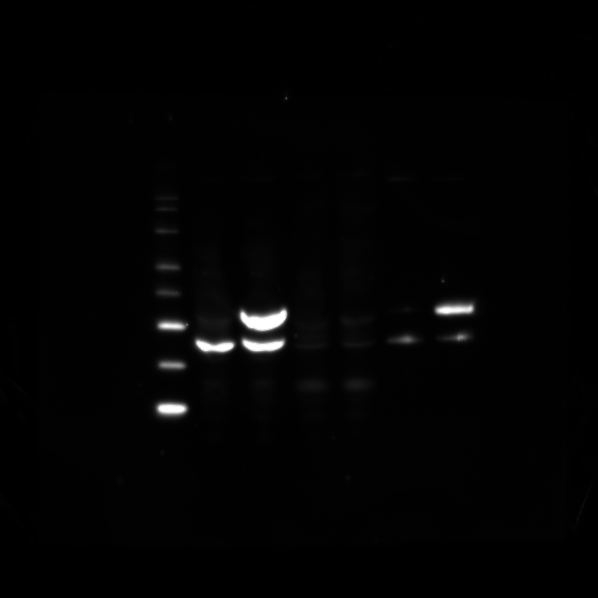

Supplement: Figure 2—source data 5. — Black arrow indicates the size of full-length MBP-295Acd, and red arrow indicates the size of the truncation product. [file elife-70236-fig2-data5.tif]

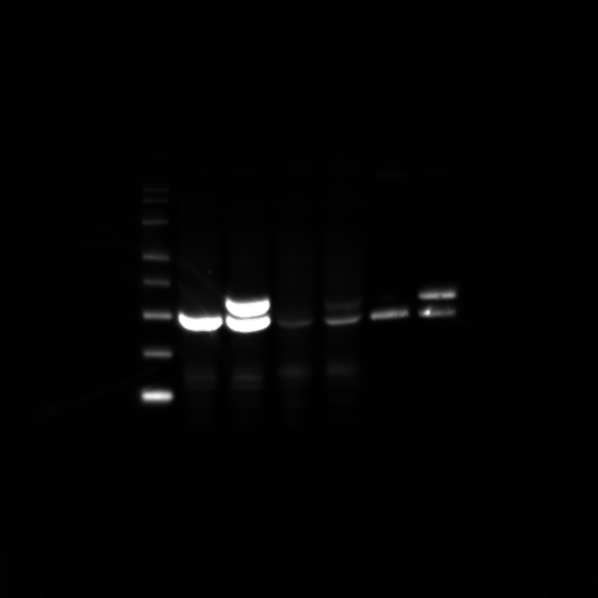

Supplement: Figure 2—source data 6. — Black arrow indicates the size of full-length MBP-322Acd, and red arrow indicates the size of the truncation product. [file elife-70236-fig2-data6.tif]
